# Supplementary material for: Preclinical Pharmacokinetics and Bioavailability of Oxypeucedanin in Rats after Single Intravenous and Oral Administration
Source: Molecules. 2022 Jun 2;27(11):3570. doi: 10.3390/molecules27113570 (PMC9182147; doi:10.3390/molecules27113570)
Supplement: Supplementary file 1 [file molecules-27-03570-s001.zip › molecules-1686872-supplementary.pdf]

Supplementary Materials

Preclinical Pharmacokinetics and Bioavailability of Oxypeucedanin in Rats after Single Intravenous and Oral Administration

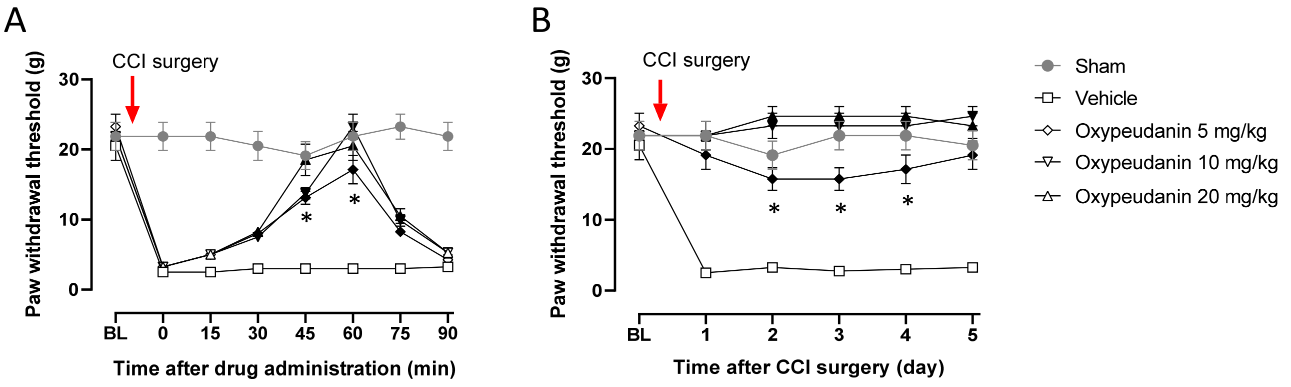

Figure S1. Oxypeucedanin dose-dependently exerts strong analgesic effects against chronic neuropathic pain model induced by chronic constricted injury (CCI) in rats. (A) Single intraperitoneal (*i.p.*) injection of oxypeucedanin 5-20 mg/kg significantly increased the paw withdrawal threshold (PWT, indicating the pain sensitivity) from 30-75 min after dosing in CCI rats ($P<0.001$), and the PWT values at 45-60 min in oxypeucedanin 20 mg/kg was higher than that of 5 mg/kg group ($P<0.05$), but was not different from 10 mg/kg group ($P>0.05$). (B) Repeated administration of oxypeucedanin 5-20 mg/kg maintained the analgesic effects for at least 5 days without trend of analgesic tolerance ($P<0.001$). The PWT values at 2-4 days in repeated treatment with oxypeucedanin 20 mg/kg was higher than that of 5 mg/kg group ($P<0.05$), but was not different from 10 mg/kg group. Sham, the sham operation group without any treatment; Vehicle, the CCI rats received vehicle treatment; Oxypeucedanin 5, 10, 20 mg/kg, the CCI rats received treatment of oxypeucedanin 5-20 mg/kg respectively. BL, baseline pain threshold before CCI surgery. The black filled points indicated significant difference compared with the corresponding data from vehicle group ($P<0.001$). Compared to oxypeucedanin 20 mg/kg group, $^*P<0.05$ (n=8). This finding indicated that oxypeucedanin 5-20 mg/kg (i.p.) can significantly attenuate the mechanical allodynia in neuropathic pain model in rats and oxypeucedanin 10 mg/kg exhibited the maximal analgesic effect in this condition.

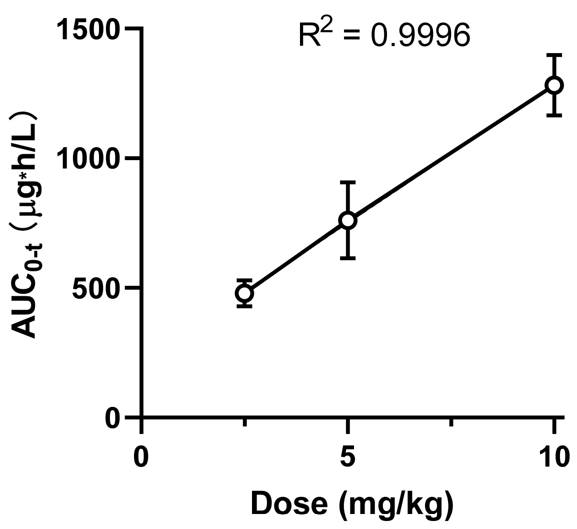

Figure S2. The area under the curve of plasma concentration-time (AUC_{0-t}) increased in a dose-proportional manner after intravenous injection of oxypeucedanin. At three dose levels of 2.5, 5, 10 mg/kg, the pharmacokinetic parameter AUC_{0-t} showed good linear correlation to dose level with the correlation coefficient (R^2) of 0.9996.

Table S1–S4: No sex differences were found in pharmacokinetic parameters of oxypeucedanin between male and female rats within the used dose levels.

Table S1. *i.v.* 2.5mg/kg.

	**male rats (n=4)**	**female rats (n=4)**
$AUC_{(0\text{-}t)}$ (μg/L*h)	454.56±93.93	500.26±244.04
$AUC_{(0\text{-}\infty)}$ (μg/L*h)	459.60±90.94	501.75±243.00
MRT (h)	0.52±0.35	0.72±0.12
$T_{1/2Z}$ (h)	0.62±0.27	0.60±0.09
V_Z (L/kg)	4.50±2.08	4.98±2.33
CL_Z (L/h/kg)	5.14±0.68	5.64±1.89
C_{2min} (μg/L)	1266.68±507.59	878.17±359.03

Table S2. *i.v.* 5mg/kg.

	**male rats (n=4)**	**female rats (n=4)**
$AUC_{(0\text{-}t)}$ (μg/L*h)	753.30±162.22	1027.19±639.99
$AUC_{(0\text{-}\infty)}$ (μg/L*h)	754.83±161.99	1029.05±694.85
MRT (h)	0.55±0.17	0.88±0.21
$T_{1/2Z}$ (h)	0.64±0.09	0.67±0.18
V_Z (L/kg)	6.28±1.20	7.50±3.32
CL_Z (L/h/kg)	6.91±1.80	10.19±6.83
C_{2min} (μg/L)	1493.17±916.49	1222.27±738.0

Table S3. *i.v.* 10mg/kg.

	**male rats (n=4)**	**female rats (n=4)**
$AUC_{(0\text{-}t)}$ (μg/L*h)	1146.95±330.94	1417.13±306.76
$AUC_{(0\text{-}\infty)}$ (μg/L*h)	1160.44±342.48	1437.39±298.04
MRT (h)	0.69±0.21	0.91±0.05
$T_{1/2Z}$ (h)	0.56±0.23	0.66±0.18
V_Z (L/kg)	6.97±1.82	7.12±3.36
CL_Z (L/h/kg)	9.16±2.49	7.21±1.68
C_{2min} (μg/L)	1822.59±579.03	1512.29±281.62

Table S4. *i.g.* 20mg/kg.

	**male rats (n=4)**	**female rats (n=4)**
$AUC_{(0\text{-}t)}$ (μg/L*h)	321.78±76.13	188.24±82.99
$AUC_{(0\text{-}\infty)}$ (μg/L*h)	378.64±73.14	217.96±105.59
MRT (h)	5.76±3.23	5.96±1.10
$T_{1/2Z}$ (h)	3.77±2.82	2.45±1.36
V_Z/F (L/kg)	285.07±227.73	319.35±29.34
CL_Z/F (L/h/kg)	54.08±9.77	105.44±43.15
C_{max} (μg/L)	76.86±54.85	53.42±10.26
